# Monitoring of Eccentric Hamstring Strength and Eccentric Derived Strength Ratios in Judokas from a Single Weight Category

**DOI:** 10.3390/ijerph19010604

**Published:** 2022-01-05

**Authors:** Jožef Šimenko, Damir Karpljuk, Vedran Hadžić

**Affiliations:** 1 Essex Pathways Department, University of Essex, Colchester CO4 3SQ, UK; 2 Faculty for Sport, University of Ljubljana, 1000 Ljubljana, Slovenia; damir.karpljuk@fsp.uni-lj.si (D.K.); vedran.hadzic@fsp.uni-lj.si (V.H.)

**Keywords:** judo, strength ratios, bilateral asymmetries, dynamic control ratio, hamstring eccentric to concentric ratio, weigh categories

## Abstract

Background: This study was designed to perform isokinetic knee testing of male judokas competing in the under 73 kg category. The main aims were: to establish the concentric (CON) and eccentric (ECC) strength profile of hamstrings (H) and CON profile of quadriceps (Q) muscles; to evaluate the differences in CON and ECC peak torques (PT) with various strength ratios and their bilateral asymmetries; the calculation of the dynamic control ratio (DCR) and H ECC to CON ratio (HEC); Methods: 12 judokas competing on a national and international levels with a mean age of 19 ± 4 years, a weight of 75 ± 2 kg and with a height of 176 ± 5 cm were tested. All the subjects were right-hand dominant. Isokinetic testing was performed on iMOMENT, SMM isokinetic machine (SMM, Maribor, Slovenia). The paired *t*-test was used to determine the difference between paired variables. The level of significance was set at *p* ≤ 0.05; Results: Statistical differences between left (L) and right (R) Q PT (L 266; R 241 Nm), H ECC PT (L 145; R 169 Nm), HQR (L 0.54; R 0.63), DCR (L 0.55; R 0.70), HEC (L 1.02; R 1.14) and PTQ/BW (L 3.57; R 3.23 Nm/kg) were shown. Bilateral strength asymmetries in CON contraction of 13.52% ± 10.04 % for Q, 10.86% ± 7.67 % for H and 22.04% ± 12.13% for H ECC contraction were shown. Conclusions: This study reports the isokinetic strength values of judokas in the under 73 kg category, emphasising eccentric hamstring strength and eccentric derived strength ratios DCR and HEC. It was shown that asymmetries are better detected using eccentric testing and that the dominant leg in judokas had stronger eccentric hamstring strength resulting in higher DCR and HEC.

## 1. Introduction

Judo represents a high-intensity and dynamic intermittent sport that requires complex skills and tactical excellence for success [1,2,3]. Studies in judo have demonstrated that adequate muscle strength is required for effective technical action [4]. Therefore, maximal muscular strength is of high importance in achieving superior physical fitness and technical supremacy in a judo bout [3,5]. Maximal strength can be measured with many devices; however, one of the most reliable methods is computerised isokinetic dynamometry [3,5,6,7,8,9]. Isokinetic diagnostic devices are used for evaluating the current condition of the locomotor system so that the strength of certain muscle groups is tested. In testing the extremities, lower concentric angular velocities are most often used for measuring maximum strength and higher concentric angular velocities (with a higher number of repetitions) for determining stamina [5]. Aside from its high reproducibility [10], isokinetic strength testing has several other significant advantages, including (1) quantification of muscle function in different contraction regimes and at different contraction velocities, (2) comparison of agonists and antagonists muscle function through various strength ratios, and (3) bilateral strength comparisons between the limbs (i.e., assessment of strength asymmetry) [7]. 

It was reported that isokinetic dynamometry could provide beneficial information regarding the detection of the strength of a particular muscle group as well as the timely detection of imbalance between muscle groups in judo [11] and as an injury prevention tool [12]. Isokinetic testing has been used in judo mainly to test knee [13,14,15,16,17,18], shoulder [19], elbow [20], trunk [21], hip [17] and ankle [14] muscle strength and strength ratios. However, the problem of these studies is that the sampling included judo athletes from multiple weight categories. It is well known that judo athletes from different weight categories have different morphological and performance characteristics [22,23,24,25,26]. Therefore, drawing conclusions from a mixed weight category sample to a particular weight category could be questionable. In addition, only two studies involving isokinetic testing in judo were done with the sample divided by the weight categories [12,27]. The first research was done by Callister et al. [27] on men’s category −95 kg and women’s −56 kg; however, weight categories changed since the 90s and the second research was done by Blach et al. [12] on all women weight categories. This shows the lack of research done in individual weight categories in male and female judo despite the research showing athletes have different performance characteristics. 

Additionally, except for the study by Malovic et al. [15], all of these studies measured only concentric (CON) strength. Judo is considered a sport where athletes are exposed to numerous concentric/concentric (CON/CON) and/or eccentric/concentric (ECC/CON) movement patterns where the opponent’s actions frequently induce the eccentric movement [28]. Additionally, ECC muscle strength is essential to optimise power in elite judo athletes, especially in producing peak lower body power during repetitiously throwing techniques [2,29]. Furthermore, the ECC training in judo is important as ECC muscle contractions enhances the performance during the concentric phase of stretch-shortening cycles and the muscles activated during lengthening movements can also function as shock absorbers, to decelerate during landing tasks or to precisely deal with high external loading [30], that is in judo usually caused by the opponent. 

Therefore, there is a great necessity in judo to measure ECC performance. This is especially true for ECC hamstring strength, which is essential for dynamic knee joint stabilization [31] and prevention of knee and, in particular, the anterior cruciate ligament (ACL) injuries, which are the most common injuries in judo (17.4%) based on the recent extensive injury analysis in judo from 2005 to 2020 on 128 top-level judo competitions [32,33]. ECC hamstring strength is used to calculate the Dynamic Control Ratio (DCR) [7,34] or, as sometimes called, the dynamic functional ratio (ECC antagonist/CON agonist) [15,35]. Additionally, it can be used to calculate the ipsilateral H_ecc_ to H_con_ ratio (HEC) [7] that has not been used in judo to the best of our knowledge. A scarce number of research studies focus on the topic of eccentric hamstring strength and its importance for judo. Based on this information, there is a clear lack of research on isokinetics in judo that would use the DCR ratio or even the HEC ratio, which has not been used. 

The aims of this study were therefore (1) to establish the CON and ECC strength profile of H and CON profile of quadriceps (Q) muscles in healthy males under 73 kg category judokas; (2) to evaluate the differences in CON and ECC peak torques (PT) with various strength ratios and their bilateral asymmetries; and (3) the calculation of the dynamic control ratio (DCR) and novel (in judo) H_ecc_ to H_con_ strength ratio (HEC).

## 2. Materials and Methods

### 2.1. Sample

In the study, we tested 12 judo athletes that are competing in the senior under 73 kg weight category on the national and international level with an age of 19 ± 3.5 years, a weight of 75 ± 1.9 kg, a height of 176 ± 4.6 cm, body mass index (BMI) of 24.4 ± 1.6, body fat 10.7 ± 4.7 % and a skeletal muscle mass of 38.4 ± 2.2 kg. Inclusion criteria were: participants competing in the senior under 73 kg weight category; participants needed to be free of acute injuries at the testing time and did not report any current musculoskeletal system pain; participants should not be involved in rapid weight loss or have a competition in the following week of the testing; otherwise, they were excluded from the study. The sample size was justified by a priori power analysis in G*power software (Version 3.1.9.6; Universität Kiel, Kiel, Germany) [36] with a type I error rate of 0.05 and 0.8 statistical power (minimum error type II). Overall, the analysis indicated that 15 participants would be sufficient to observe significant large-sized acute effects (Cohen’s d = 0.80). However, we were able to evaluate 12 judokas representing 52% of male athletes who participated in the national championships, which has put our actual statistical power at 0.71.

Hand dominance was used as an indicator of judo dominance, where the participants were asked which hand they used to write, draw, and throw a ball, as previously used [37]. All were right-hand dominant judokas, meaning that they are mostly (depending on the fight tactics) right stance dominant fighters, where the left leg is the supporting leg/non-dominant and the right leg is the attacking/dominant leg. They have been training judo for 10 ± 4 years. At the time of the testing, judokas had, on average 1. DAN belt degree (black belt) ±0.78. The Faculty of Sport, University of Ljubljana Ethical Board (No. 2033/2013) approved the study. During the study, the principles outlined in the Declaration of Helsinki were followed. Upon recruitment, a signed informed consent form was obtained from participants parents or guardians to participate in the study voluntarily. 

### 2.2. Experimental Procedure and Data Analysis

Anthropometric measurements were taken in the morning between 9 am and 12 am. Body height was measured with a stadiometer (GPM, Zürich, Switzerland). Body composition measurements were performed using bioelectrical impedance analysis with the InBody 720 Tetrapolar 8-Point Tactile Electrode System (Biospace Co., Ltd., Seoul, South Korea). The InBody 720 apparatus utilises the technology for measuring body composition by using the method of Direct Segmental Multi-Frequency Bioelectrical Impedance Analysis. With InBody 720, we measured body weight, BMI, skeletal muscle mass and body fat mass.

Afterwards, isokinetic testing took place. It was performed by the same examiner with extensive isokinetic testing experience in the Laboratory for isokinetic testing at the Faculty of Sport in Ljubljana, Slovenia. Laboratory was air-conditioned and the room temperature was held between 22–24 °C. Testing was performed between 10 AM and 4 PM over one week. Each testing session started with a warm-up consisting of cycling for 6 min at a moderate pace (50–100 W), followed by a 15 s stretch of Q and H. All participants were given a detailed explanation about the testing procedure. 

**Figure 1:**
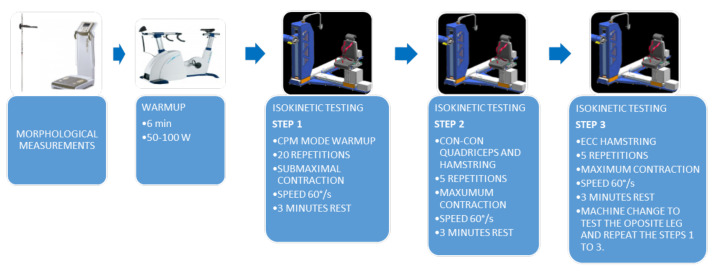
Flowchart of the testing protocol.

Testing was performed for quadriceps and hamstrings in concentric and also for hamstring in eccentric mode. The iMoment, SMM isokinetic dynamometer (SMM, Maribor, Slovenia) was used for testing [38,39,40]. The device was calibrated, and the gravity correction was executed according to the manufacturer’s procedures. Judokas were tested in a sitting position. Forward sliding on the seat was prevented using 4-point belts that pushed the pelvis downward and backwards but were not uncomfortable for the participants and also controlled the trunk movement. The thigh of the tested leg was secured using a special additional belt. During testing, the subjects were instructed to hold the side handles at the chair’s base. The knee joint rotation axis was identified through the lateral femoral condyle and aligned with the motor axis. A range of motion of 60° was set from 90° to 30° knee flexion (full flexion considered 0) [41]. Testing was performed at 60°/s for both H and Q in concentric and H eccentric contraction modes. Gravity error torque was recorded for every subject. Before testing, each participant performed a series of 20 submaximal repetitions at a given velocity in a continuous passive mode (CPM) followed by a 3 min break. After the initial CPM set, each participant performed 5 maximal contractions in the following order: (1) five consecutive concentric Q and H contractions followed by a 60 s pause, (2) five eccentric H contractions. When testing of one side was completed, a 3-min break followed, during which the machine setting was changed to accommodate for the opposite leg. The first tested leg was assigned randomly for each subject. There was no verbal coaching during testing repetitions. The main outcome measure was peak torque (PT) which was later normalised for body weight (BW) and expressed as PT/BW as suggested by Jaric et al. [42]. Following strength ratios were also calculated: the concentric H/Q ratio (HQR), H_ecc_/Q_con_ ratio—the dynamic control ratio—DCR [34], and H_ecc_/H_con_ ratio (HEC) [7]. Finally, we calculated the relative strength difference between left (L) and right (R) leg for all testing conditions—a neuromuscular measure known in the literature as the bilateral strength asymmetry [43] using the following formula: [1 − (PT L/PT R)] × 100. The bilateral strength asymmetry expressed in percentages was used regardless of which leg was stronger. The testing protocol is shown in Figure 1. 

### 2.3. Statistical Analysis

Data were processed and presented using the SPSS for Windows 27.0 statistical package (SPSS, Inc., Chicago, IL, USA). The Shapiro–Wilk test was used to assess the normality of the data. To determine differences in paired variables, a paired T-test was used with a statistical significance set at *p* ≤ 0.05. Effect sizes (ES) were calculated utilizing Cohen’s d. Threshold values for ES statistics were: >0.2 small, >0.5 moderate, >0.8 large, >1.3, very large [44].

## 3. Results

The results of isokinetic testing for L (non-dominant side) and R (dominant side) H and Q in both CON and ECC modes are presented in Table 1. Judokas in the weight category under 73 kg statistically differ in L and R Q peak torque (PT-Q) t(11) = 2.47, *p* = 0.031; L and R H ECC peak torque (PT-H-ecc) t(11) = 3.33, *p* = 0.007; L and R H to Q ratio—HQR t(11) = 4.02, *p* = 0.002.; L and R dynamic control ratio DCR t(11) = 7.05, *p* = 0.001; L and R H ECC to H CON ratio HEC t(11) = 3.31, *p* = 0.007 and L and R Q peak torque to body weight PTQ/BW t(11) = 2.52, *p* = 0.029. Other variables did not statistically differ. 

Bilateral strength asymmetries are presented in Table 2. The Q bilateral strength asymmetry in the concentric mode was 14%, in H concentric mode was 11% and in H eccentric mode was 22% between L and R leg.

## 4. Discussion

Our results demonstrate the CON and ECC H and CON Q strength profile in healthy males under the 73 kg category. Furthermore, the tested legs’ HQR, DCR and HEC ratios were calculated and bilaterally compared. Strength ratios showed significant bilateral asymmetries favouring the right (attacking) leg. Our study is the first to report the ipsilateral HEC ratio for hamstrings in judokas; only the second one [15] in judo to use eccentric isokinetic measurements in the knee joint and only the 3rd one [12,27] to conduct a study with isokinetic testing on a sample of a selected weight category. 

### 4.1. Isokinetic Absolute Values

Our data show that judokas competing in the under 73 kg weight category achieved absolute PT values of 266 ± 31 Nm on the L and 241 ± 40 Nm on the R Q and 142 ± 16 Nm on the L and 148 ± 12 Nm on the R H. Values from L and R H PT did not statistically differ. In contrast, Q PT values showed a significant difference (*p* = 0.031) with greater values in the L Q. Compared to a study with senior Serbian judokas, who achieved values of 271 Nm on the R Q and 271 Nm on the L Q, and the 109 Nm on the R H and 110 Nm on the L H [5], our judokas have achieved lower absolute PT values for Q, while they achieved much better absolute PT values for H. Similar absolute Q PT values for the L and R leg (L 243 Nm; R 240 Nm) in senior judokas were reported by Malovic et al. [15], with higher absolute H PT values (L 162 Nm; R 152 Nm). However, such comparisons should be taken with caution as the sample of the aforementioned studies included judokas from all weight categories (bodyweight of the sample [5] 81.02 ± 42.98 kg; [15] 82.30 ± 17.03 kg). Therefore, possible characteristics of a particular weight category can be hidden in the average of all participants. Additionally, reporting and comparing absolute values on a weight mixed sample for a weight category sensitive sport can be especially problematic and misleading. Therefore, research in judo should emphasize sampling from particular weight categories and report PT as absolute and relative values. Overall, our judokas achieved a good absolute CON H PT. 

### 4.2. Isokinetic Relative Values

Relative Q peak torques values (PTQ/BW) of our study (L 3.57 ± 0.59; R 3.23 ± 0.53) are similar to the study done by Lech et al. [4], where mean relative Q peak torque of 3.44 ± 0.59 Nm/kg was reported in the sample of 7 athletes. However, higher differences occur when we compare flexors data with 1.77 ± 0.58 Nm/kg to our 1.90 ± 0.23 Nm/kg on the L and 1.99 ± 0.17 Nm/kg on the R side. Again, comparing these results can be problematic as the comparison study sample comprises judokas from several weight categories (mean bodyweight 82.2 ± 7.6 kg), so athletes from higher weight categories can negatively influence the mean representation of the data. Therefore, the need for testing athletes from a particular weight category is even more critical. Normative values recommend that athletes, when tested at the 60°/s, should be between 2.7–3.2 Nm/kg in Q PT and between 1.6–2.0 Nm/kg in H PT [9,45]. Our athletes’ relative Q PT/BW values are above, while our H PT/BW values are well in line with the recommended values. Furthermore, high maximum relative H torques were reported to highly correlate with the judo activity index (r = 0.76, respectively) [4], highlighting the importance of hamstring strength in judo. 

### 4.3. Strength Ratios

Measuring ECC H PT allowed us to calculate the HEC ratio between H ECC and H CON values for the first time in judo. Maybe we should stress that, unlike concentric strength, the eccentric muscle strength is more difficult to normalize to body weight, as it is strongly associated with the neurological component, making comparisons between studies difficult; therefore, the HEC ratio is much easier to follow and compare. Our athletes reported HEC values of 1.02 ± 0.08 for the L and 1.14 ± 0.15 for the R leg. The HEC ratio over 1 shows that the ECC strength of the H is greater than the CON strength, as it is expected with a normal muscle force-velocity relationship (e.g., normal Hill’s graph). Our data suggest that the H ECC strength is higher from CON for 2% in the L and 14% on the R leg. Values on the R leg are in line with the values from other sports like volleyball, which are around 10% [7] higher for ECC H strength. However, the values for the L leg with just 2% show a low ECC H strength of the supporting leg for the right stance dominant judokas. Moreover, the HEC ratio showed significant differences between L and R leg (*p* = 0.007). 

The most significant differences in the L to the R leg are shown in ECC H PT, with statistically (*p* = 0.007) lower values on the L leg. The same statistical difference is also shown in the dynamic control ratio-DCR on the L leg (L 0.55; R 0.70), which according to some authors [9], could present an increased risk of an H strain. Therefore, measuring ECC H strength in judo is of great importance as it helps to calculate the DCR ratio. In judo, a DCR ratio on a mixed weight sample was reported in for L and R leg for seniors (L 0.71 ± 0.16; R 0.72 ± 0.12), juniors (L 0.81 ± 0.28; R 0.78 ± 0.25) and cadets (L 0.84 ± 0.10; R 0.91 ± 0.24) [15]. In comparison, we can see that the DCR is similarly lower on the L leg in senior and cadets compared to our athletes, which indicates the low ECC H strength of the supporting leg for the right stance dominant fighters. In other sports like sprinting and soccer, a DCR of 1.0 has been proposed [46]. Furthermore, DCR acts as a predictor that indicates a significant capacity of the H to provide joint stabilisation during knee extension [47]. This indicates that eccentrically acting H has the ability to break the action of the concentrically contracting Q, meaning that this helps reduce the tibia’s anterior displacement on the femur and prevent hyperextension of the knee [46], thus protecting overstraining of ACL. The knee is the most frequently injured site in judo [32], with an ACL tear being the most frequent cause [33]. The ACL is also the most serious knee injury in judo [48]. Therefore, suggestions have been made for the importance of developing an exercise prevention programme for judo [48,49,50]. Combined with the injury preventive programmes, the DCR ratio with the eccentric H strength could also help in the early identification of vulnerable judokas and optimal conditioning of judo athletes. It should be stressed that some professionals are anxious to apply eccentric strength testing as we have noticed a study by Ermiş et a [13], where they reported that their study was conducted with concentric contractions alone to prevent injuries of the H muscles in subjects. This is unreasonable as the ECC testing of hamstrings is safe for a healthy athlete and there was only one report of a hamstring muscle strain caused by isokinetic testing [51], where testing for performed on an athlete with a known hamstring strain. Eccentric hamstring strength testing also does not take excessive additional time. If we add a 60-s break after the Q and H CON/CON test and 5 ECC H repetitions that last 10 s, we extend the testing for just 70 s. However, the additional data from ECC H PT could be of great use for calculating the DCR and HEC ratios. However, we must acknowledge that isokinetic dynamometers and testing are expensive [52] and time-consuming [53] for some athletes and clubs. Therefore, we can recommend a two-stage screening approach. The first one would be the use of field screening with the Single Leg Hamstring Bridge Test (SLHBT) [52]. The SLHBT is a simple and affordable test that could be used in the initial physical conditioning screening of hamstring strength and asymmetry evaluation [54,55]. If the asymmetries exceed 15% [56], we recommend the second stage in which the isokinetic testing should comprehensively evaluate those athletes. We recommend that national judo associations and coaches use this approach to tackle the high rates of knee joint injuries. Additionally, a high CON PT of Q and a low CON PT of H results in a low HQR ratio (L 0.54; R 0.63), which puts the knee joint at risk. It was reported that lower than 60% of HQR is associated with non-contact leg injuries [57]. This could be explained by all judokas being right-hand dominant, which usually reflects in the judo bout to be right stance dominant. In this position, the right stance dominant judokas usually operate on the L leg as a supporting leg, and the R leg is used as the “execution” or attacking leg. Therefore, the execution leg (in our case, the right leg) is getting much more concentric and eccentric work than the supporting leg, which could lead to imbalances between muscle groups. Especially if the judokas training is primarily unilateral and they perform their throwing techniques only to their dominant side [58]. These circumstances often occur when judokas train their Tokui waza—a special technique. Standing-supporting leg hamstring is therefore at a higher risk of being injured, especially when judoka is attacking or being attacked with the uchi-mata technique, which is one of the most used techniques and the standing leg hamstring gets stretched to the limits. Furthermore, sudden knee extension of the pivoting leg with the hip flexed while executing the uchi-mata move can cause a rupture of the hamstring tendon [59]. Another H injury mechanism can occur to the supporting leg while being counterattacked by the opponent with overturning while executing the tai otoshi throw [59]. In this position, the ECC H strength of the standing leg plays a vital role in preventing H injuries. Moreover, a high CON and ECC H strength can additionally protect the knee joint from injuries like ACL injuries and before H injuries or re-injuries [60,61,62,63].

Unilateral training can lead to imbalances in muscle strength which increase the occurrence of injuries. We may notice that concentric strength differences were within the proposed 15% limit [9], while eccentric strength asymmetry was 22% showing more sensitivity of eccentric strength testing to detect strength differences. Our results highlight the importance of bilateral movement development and good throwing techniques to the dominant and non-dominant body sides. This will not only help lower functional asymmetries, but it will also aid in a symmetrical morphological development, and as it was shown, good bilateral throw performance helps achieve greater competition performance all year round [64].

We acknowledge some limitations of this study. For even greater accuracy, the sample of the study could be divided by age groups. Therefore, we recommend conducting isokinetic testing within selected weight categories of the selected age group for further studies. Further research can also be recommended for females and their weight categories in selected age groups. Additionally, our athletes were in the preparation period, and there is a chance that their strength and conditioning was not at their peak; however, the PT showed acceptable values. We did not collect any previous injury data, which could give us better insight into the importance of the new HEC ratio. Therefore, we recommend further studies to account for injury history additionally. This is the first study that calculated the HEC ratio in judo, limiting the findings’ discussion. Moreover, the athletes tokui-waza (prefered techniques) is not known to us and if it is executed bilaterally or unilaterally, this can significantly impact the body strength imbalances. Therefore, we recommend further studies to account for this information.

## 5. Conclusions

This study reports the isokinetic strength values of judokas in the under 73 kg category, emphasising eccentric hamstring strength and eccentric derived strength ratios DCR and HEC. It was shown that asymmetries are better detected using eccentric testing and that the dominant leg in judokas had stronger eccentric hamstring strength resulting in higher DCR and HEC. Our data also suggest that special attention should be put on the supporting leg and its ECC H strength. However, more studies are needed to establish reliable reference values and evaluate the potential association of eccentric hamstring strength with knee injuries in judo and its potential for training optimisation.

## Figures and Tables

**Table 1:** Mean (±SD) values for L and R quadriceps (Q) and hamstring (H) concentric and hamstring eccentric (PTHecc) peak torques (PT), PT per kg of weight (PT/BW) and strength ratios (HQR, DCR and HEC) at 60°/s with paired *t*-test.

	Group									
	Left Side (Non-Dominant)		Right Side (Dominant)		95% CI					
Variables	Mean	SD	Mean	SD	Lower	Upper	*df*	*t*	*p*	*ES*
PT-Q (Nm)	266	31	241	40	2.80	48.36	11	2.47	0.031 *	0.714
PT-H (Nm)	142	16	148	12	−16.78	4.78	11	1.23	0.246	0.357
PT-H-ecc (Nm)	145	21	169	30	−40.42	−8.25	11	3.33	0.007 *	0.961
HQR	0.54	0.06	0.63	0.09	−0.14	−0.04	11	4.02	0.002 *	1.159
DCR	0.55	0.08	0.70	0.09	−0.21	−0.11	11	7.05	0.001 *	2.035
HEC	1.02	0.08	1.14	0.15	−0.20	−0.04	11	3.34	0.007 *	0.964
PTQ/BW (Nm/kg)	3.57	0.43	3.23	0.53	0.04	0.65	11	2.52	0.029 *	0.726
PTH/BW (Nm/kg)	1.90	0.23	1.99	0.17	−0.23	0.06	11	1.29	0.223	0.373

* *p* ≤ 0.05; Legend: PT—peak torque; Q—quadriceps muscles, H—hamstring muscles; Hecc—H eccentric mode data; HQR—hamstring/quadriceps ratio; DCR—dynamic control ratio; HEC—H eccentric contraction divided by H concentric contraction ratio; PTQ/BW—peak Q torque divided by body weight; PTH/BW (Nm/kg)—peak H torque divided by body weight; ES—effect size; df—degrees of freedom; CI—confidence interval.

**Table 2:** Bilateral strength asymmetry (%) of quadriceps (Q) and hamstring (H) muscles in under 73 kg judokas.

Variables of Asymmetry	Mean (%)	SD	95% Confidence Interval
Q in concentric mode	13.52%	10.04	(7.14–19.90)
H in concentric mode	10.86%	7.67	(5.98–15.72)
H in eccentric mode	22.04%	12.13	(14.33–29.74)

Legend: Q—quadriceps muscles, H—hamstring muscles; SD—standard deviation.

## Data Availability

The data that support the findings of this study are available from the corresponding author upon reasonable request.

## References

[1] Callister R., Callister R.J., Staron R.S., Fleck S.J., Tesch P., Dudley G.A. (1991). Physiological characteristics of elite judo athletes. Int. J. Sports Med..

[2] Franchini E., Del Vecchio F.B., Matsushigue K.A., Artioli G.G., Del F.B., Matsushigue K.A., Artioli G.G., Del Vecchio F.B., Matsushigue K.A., Artioli G.G. (2011). Physiological profiles of elite judo athletes. Sport. Med..

[3] Ghrairi M., Hammouda O., Malliaropoulos N. (2014). Muscular strength profile in Tunisian male national judo team. Muscles. Ligaments Tendons J..

[4] Lech G., Chwała W., Ambrozy T., Sterkowicz S. (2015). Muscle torque and its relation to technique, tactics, sports level and age group in judo contestants. J. Hum. Kinet..

[5] Drid P., Drapsin M., Trivic T., Bratic M., Obadov S. (2010). Thigh muscles flextion/extension ratio in elite judo players. J. Combat Sport. Martial Arts..

[6] Andrade M.D.S., De Lira C.A.B., Koffes F.D.C., Mascarin N.C., Benedito-Silva A.A.A., Da Silva A.C. (2012). Isokinetic hamstrings-to-quadriceps peak torque ratio: The influence of sport modality, gender, and angular velocity. J. Sports Sci..

[7] Hadzic V., Sattler T., Markovic G., Veselko M., Dervisevic E. (2010). The isokinetic strength profile of quadriceps and hamstrings in elite volleyball players. Isokinet. Exerc. Sci..

[8] Almosnino S., Stevenson J.M., Bardana D.D., Diaconescu E.D., Dvir Z. (2012). Reproducibility of isokinetic knee eccentric and concentric strength indices in asymptomatic young adults. Phys. Ther. Sport.

[9] Dervišević E., Hadžić V. (2012). Quadriceps and hamstrings strength in team sports: Basketball, football and volleyball. Isokinet. Exerc. Sci..

[10] Kannus P. (1994). Isokinetic evaluation of muscular performance: Implications for muscle testing and rehabilitation. Int. J. Sports Med..

[11] Drid P., Vujkov S., Trivic T., Ostojic S.M., Stojanovic M., Purkovic S., Vujkov S., Purkovic S., Trivic T., Stojanovic M. (2011). Physiological adaptations of a specific muscleimbalance reduction training programme in the elite female judokas. Arch. Budo.

[12] Blach W., Drapsin M., Lakicevic N., Bianco A., Gavrilovic T., Roklicer R., Trivic T., Cvjeticanin O., Drid P., Kostrzewa M. (2021). Isokinetic Profile of Elite Serbian Female Judoists. Int. J. Environ. Res. Public Health.

[13] Ermiş E., Yilmaz A.K., Kabadayi M., Bostanci Ö., Mayda M.H. (2019). Bilateral and ipsilateral peak torque of quadriceps and hamstring muscles in elite judokas. J. Musculoskelet. Neuronal Interact..

[14] Ghram A., Young J.D., Soori R., Behm D.G. (2019). Unilateral Knee and Ankle Joint Fatigue Induce Similar Impairment to Bipedal Balance in Judo Athletes. J. Hum. Kinet..

[15] Malovic P., Bjelica D., Atanasov D., Trivic T., Drapsin M., Trajkovic N., Maksimovic N., Drid P. (2020). Knee strength ratios in male judokas: Age-related differences. Arch. Budo.

[16] Coban O., Yildirim N.U., Yasa M.E., Akinoglu B., Kocahan T. (2021). Determining the number of repetitions to establish isokinetic knee evaluation protocols specific to angular velocities of 60°/second and 180°/second. J. Bodyw. Mov. Ther..

[17] Lee J.B., Kim Y.K. (2020). The Relationship between Dynamic Balance Ability and Low Limb Isokinetic Muscle Function of Elite Judo Athletes. Asian J. Kinesiol..

[18] Struzik A., Pietraszewski B. (2015). Lower limb torque asymmetry in judo competitors evaluated in isokinetic conditions. Med. Sport.

[19] Marcondes F.B., Castropil W., Schor B., Miana A., Vasconcelos R., Etchebehere M. (2019). Shoulder isokinetic performance in healthy professional judo athletes: Normative data. Acta Ortopédica Bras..

[20] Ichinose Y., Kanehisa H., Ito M., Kawakami Y., Fukunaga T. (1998). Morphological and functional differences in the elbow extensor muscle between highly trained male and female athletes. Eur. J. Appl. Physiol..

[21] Kim M.S., Kim S.H., Bang H.S. (2017). The Change of Isokinetic Strength and Anaerobic Exercise Performance in Judo Athletes Depending on the Set Interval During Weight Training. J. Korean Alliance Martial Arts..

[22] Chaabene H., Negra Y., Bouguezzi R., Capranica L., Franchini E., Prieske O., Hbacha H., Granacher U. (2018). Tests for the assessment of sport-specific performance in Olympic combat sports: A systematic review with practical recommendations. Front. Physiol..

[23] Detanico D., Budal Arins F., Dal Pupo J., Dos Santos S.G., Arins F.B., Pupo J.D., Santos S.G.D.O.S., Dal Pupo J., Dos Santos S.G., Budal Arins F. (2012). Strength Parameters in Judo Athletes: An Approach Using Hand Dominance and Weight Categories. Hum. Mov..

[24] Martins F.P., de Souza L.S.D.P., de Campos R.P., Bromley S.J., Takito M.Y., Franchini E. (2019). Techniques utilised at 2017 Judo World Championship and their classification: Comparisons between sexes, weight categories, winners and non-winners. Ido Mov. Cult..

[25] Shavkatovich F.A. (2020). The relationship between the weight classes and competitive activity of judo athletes. Int. J. Phys. Educ. Sport. Health.

[26] Sterkowicz-Przybycień K., Miarka B., Fukuda D.H. (2017). Sex and Weight Category Differences in Time-Motion Analysis of Elite Judo Athletes: Implications for Assessment and Training. J. Strength Cond. Res..

[27] Callister R., Callister R.J., Fleck S.J., Dudley G.A. (1990). Physiological and performance responses to overtraining in elite judo athletes. Med. Sci. Sports Exerc..

[28] Mackala K., Witkowski K., Vodičar J., Šimenko J., Stodółka J. (2019). Acute effects of speed-jumping intervention training on selected motor ability determinants: Judo vs. soccer. Arch. Budo.

[29] Harris D.M., Foulds S., Latella C. (2019). Evidence-Based Training Recommendations for the Elite Judoka. Strength Cond. J..

[30] Vogt M., Hoppeler H.H. (2014). Eccentric exercise: Mechanisms and effects when used as training regime or training adjunct. J. Appl. Physiol..

[31] Ayala F., Sainz de Baranda P., De Ste Croix M., Santonja F. (2012). Absolute reliability of five clinical tests for assessing hamstring flexibility in professional futsal players. J. Sci. Med. Sport.

[32] Błach W., Smolders P., Rydzik Ł., Bikos G., Maffulli N., Malliaropoulos N., Jagiełło W., Maćkała K., Ambroży T. (2021). Judo Injuries Frequency in Europe’s Top-Level Competitions in the Period 2005–2020. J. Clin. Med..

[33] Koshida S., Deguchi T., Miyashita K., Iwai K., Urabe Y. (2010). The common mechanisms of anterior cruciate ligament injuries in judo: A retrospective analysis. Br. J. Sports Med..

[34] Dvir Z., Eger G., Halperin N., Shklar A. (1989). Thigh muscle activity and anterior cruciate ligament insufficiency. Clin. Biomech..

[35] Cozette M., Leprêtre P.-M., Doyle C., Weissland T. (2019). Isokinetic Strength Ratios: Conventional Methods, Current Limits and Perspectives. Front. Physiol..

[36] Faul F., Erdfelder E., Lang A.-G., Buchner A. (2007). G*Power 3: A flexible statistical power analysis program for the social, behavioral, and biomedical sciences. Behav. Res. Methods.

[37] Mikheev M., Mohr C., Afanasiev S., Landis T., Thut G. (2002). Motor control and cerebral hemispheric specialization in highly qualified judo wrestlers. Neuropsychologia.

[38] Kambič T., Lainščak M., Hadžić V. (2020). Reproducibility of isokinetic knee testing using the novel isokinetic SMM iMoment dynamometer. PLoS ONE.

[39] Križaj J., Rauter S., Vodičar J., Hadžić V., Šimenko J. (2019). Predictors of vertical jumping capacity in soccer players. Isokinet. Exerc. Sci..

[40] Hadžić V., Širok B., Malneršič A., Čoh M. (2019). Can infrared thermography be used to monitor fatigue during exercise? A case study. J. Sport Health Sci..

[41] Dervišević E., Hadžić V., Karpljuk D., Radjo I. (2006). The influence of different ranges of motion testing on the isokinetic strength of the quadriceps and hamstrings. Isokinet. Exerc. Sci..

[42] Jaric S., Mirkov D., Markovic G. (2005). Normalizing physical performance tests for body size: A proposal for standardization. J. strength Cond. Res. / Natl. Strength Cond. Assoc..

[43] Barber S.D., Noyes F.R., Mangine R.E., McCloskey J.W., Hartman W. (1990). Quantitative assessment of functional limitations in normal and anterior cruciate ligament-deficient knees. Clin. Orthop. Relat. Res..

[44] Sullivan G.M., Feinn R. (2012). Using Effect Size—or Why the P Value Is Not Enough. J. Grad. Med. Educ..

[45] Šimenko J. (2017). Upotreba izokinetike kao metode u treningu i rehabilitaciji sportaša. Kond. Trening.

[46] Coombs R., Garbutt G. (2002). Developments in the use of the hamstring/quadriceps ratio for the assessment of muscle balance. J. Sport. Sci. Med..

[47] Carvalho A., Brown S., Abade E. (2016). Evaluating injury risk in first and second league professional Portuguese soccer: Muscular strength and asymmetry. J. Hum. Kinet..

[48] Akoto R., Lambert C., Balke M., Bouillon B., Frosch K.-H.H., Höher J. (2018). Epidemiology of injuries in judo: A cross-sectional survey of severe injuries based on time loss and reduction in sporting level. Br. J. Sports Med..

[49] Malliaropoulos N.G., Callan M., Johnson J., Frizziero L. (2019). Comprehensive training programme for judo players nine plus 9+: Possible lower limb primary injury prevention. Muscle Ligaments Tendons J..

[50] Von Gerhardt A.L., Vriend I., Verhagen E., Tol J.L., Kerkhoffs G.M.M.J.M.J., Reurink G. (2020). Systematic development of an injury prevention programme for judo athletes: The IPPON intervention. BMJ Open Sport Exerc. Med..

[51] Orchard J., Steet E., Walker C., Ibrahim A., Rigney L., Houang M. (2001). Hamstring muscle strain injury caused by isokinetic testing. Clin. J. Sport Med. Off. J. Can. Acad. Sport Med..

[52] Freckleton G., Cook J., Pizzari T. (2014). The predictive validity of a single leg bridge test for hamstring injuries in Australian rules football players. Br. J. Sports Med..

[53] Dvir Z. (2000). Isokinetic Muscle Testing: Reflections on Future Venues. Hong Kong Physiother. J..

[54] Mahnič N., Rauter S., Hadžić V., Šimenko J. (2021). The Single Leg Bridge Test (SLBT) as a field test to measure hamstring strength in young footballers. Sci. Sport..

[55] Pori P., Kovčan B., Vodičar J., Dervišević E., Karpljuk D., Hadžić V., Šimenko J. (2021). Predictive Validity of the Single Leg Hamstring Bridge Test in Military Settings. Appl. Sci..

[56] Kyritsis P., Bahr R., Landreau P., Miladi R., Witvrouw E. (2016). Likelihood of ACL graft rupture: Not meeting six clinical discharge criteria before return to sport is associated with a four times greater risk of rupture. Br. J. Sports Med..

[57] Kim D., Hong J. (2011). Hamstring to quadriceps strength ratio and noncontact leg injuries: A prospective study during one season. Isokinet. Exerc. Sci..

[58] Šimenko J. (2012). Analysis of movement efficiency of judoists. Šport Rev. Za Teor. Prakt. Vprasanja Sport..

[59] Kurosawa H., Nakasita K., Nakasita H., Sasaki S., Takeda S. (1996). Complete avulsion of the hamstring tendons from the ischial tuberosity. A report of two cases sustained in judo. Br. J. Sports Med..

[60] Guelich D.R., Xu D., Koh J.L., Nuber G.W., Zhang L.-Q. (2016). Different roles of the medial and lateral hamstrings in unloading the anterior cruciate ligament. Knee.

[61] Mias E., Starrs P. (2014). The effect of a six week eccentric hamstring strengthening protocol on anterior tibio-femoral translation. Br. J. Sports Med..

[62] Biscarini A., Botti F.M., Pettorossi V.E. (2013). Selective contribution of each hamstring muscle to anterior cruciate ligament protection and tibiofemoral joint stability in leg-extension exercise: A simulation study. Eur. J. Appl. Physiol..

[63] Schmitt B., Tim T., McHugh M. (2012). Hamstring injury rehabilitation and prevention of reinjury using lengthened state eccentric training: A new concept. Int. J. Sports Phys. Ther..

[64] Šimenko J., Hadžič V., Had V. Bilateral Throw Execution in Young Judokas for a Maximum All Year Round Result. Int. J. Sports Physiol. Perform..

